# Reducing Quorum Sensing-Mediated Virulence Factor Expression and Biofilm Formation in *Hafnia alvei* by Using the Potential Quorum Sensing Inhibitor L-Carvone

**DOI:** 10.3389/fmicb.2018.03324

**Published:** 2019-01-09

**Authors:** Tingting Li, Yongchao Mei, Binbin He, Xiaojia Sun, Jianrong Li

**Affiliations:** ^1^Key Laboratory of Biotechnology and Bioresources Utilization (Dalian Minzu University), Ministry of Education, Dalian, China; ^2^College of Food Science and Technology, Bohai University, Jinzhou, China; ^3^National & Local Joint Engineering Research Center of Storage, Processing and Safety Control Technology for Fresh Agricultural and Aquatic Products, Jinzhou, China

**Keywords:** *Hafnia alvei*, L-carvone, quorum sensing inhibitor, *in silico* analysis, RT-qPCR

## Abstract

Quorum sensing (QS), one of the most remarkable microbiological discoveries, is considered a global gene regulatory mechanism for various traits in bacteria, including virulence and spoilage. *Hafnia alvei*, an opportunistic pathogen and a dominant psychrophile, uses the *lux*-type QS system to regulate the production of virulence factors and biofilms, which are harmful to the food industry. Based on the QS interference approach, this study aimed to reveal the efficacy of L-carvone at sublethal concentrations on QS-regulated virulence factors and biofilm formation in *H. alvei.* QS inhibitory activity was demonstrated by the reduction in swinging motility (61.49%), swarming motility (74.94%), biofilm formation (52.41%) and acyl-homoserine lactone (AHL) production (0.5 μL/mL). Additionally, *in silico* analysis and RT-qPCR studies for AHL synthase HalI and QS transcriptional regulator HalR revealed a plausible molecular mechanism for QS inhibition by L-carvone. These findings suggest that L-carvone (a main component of spearmint essential oils) could be used as a novel quorum sensing inhibitor to control *H. alvei* in the food industry.

## Introduction

*Hafnia alvei* is a Gram-negative, facultatively anaerobic, rod-shaped, motile bacterium of the family *Enterobacteriaceae*; it is an opportunistic pathogen and a dominant psychrophile found in putrid food (Vivas et al., 2008). It has been widely isolated from different food products, such as raw meat, dairy and aquatic products, and specially from various packed food products stored at low temperatures (Kennedy et al., 2010; Chen et al., 2011; Tan et al., 2014). Based on these characteristics, *H. alvei* is often considered a specific spoilage organism (SSO) that causes severe nutrition and safety problems in these food matrices by producing extracellular enzymes and siderophores, and forming biofilms. Recent studies have described the key roles of virulence factors and biofilm production in *H. alvei*, which is regulated by quorum sensing (QS) systems (Viana et al., 2009; Hou et al., 2017b).

Quorum sensing is a process that allows single-cell organisms (like bacteria) cooperate, communicate, and act collectively. By this process, they can produce, release, detect, and establish connections with small chemical molecules called autoinducers, which in Gram-negative bacteria are acyl-homoserine lactones (AHLs). Thus, AHL-mediated QS systems are usually composed of the LuxI-type autoinducer synthetase, and cytoplasmic LuxR-type proteins, which are receptors activated by AHLs (Ng and Bassler, 2009). Among these two proteins, LuxR-type proteins have more complex functions. With the increase of bacterial density (achieving a certain threshold), the ligand-binding domain of the LuxR-type proteins will bind with AHLs, which will cause changes in the protein conformation and stimulate the formation of AI-LuxR compound proteins, and lead to the binding of the DNA-binding domain of these compound proteins to target genes, thereby regulating the expression of bacterial multiplex phenotypic features such as virulence factors and biofilms (Almeida et al., 2016). Therefore, interfering with AHL-mediated QS systems by using certain compounds, generally called QS inhibitors (QSIs), may be a better strategy to prevent bacterial food spoilage. Compared to antibiotics and antiseptics, QSIs aim to make the bacteria ‘surrender’ instead of killing them, which would weaken them from having resistance (Defoirdt, 2017).

Nowadays, many synthetic and natural products have been called QSIs; however, only a few of them have a therapeutic value, due to the instability or high toxicity of most other compounds (Defoirdt et al., 2013). Due to the property of low toxicity, some natural compounds from spice plants have been widely used as antimicrobial agents in the food industry, such as curcumin, vanillin, menthol, and cinnamaldehyde (Fitzgerald et al., 2003; Husain et al., 2015; Ding et al., 2017). Therefore, extracting natural compounds from spice plants to obtain effective QSIs has become a promising research hotspot. L-Carvone (or (*R*4)-(-)-carvone), a monoterpene, is the main component of spearmint essential oils from traditional spice plants and medical herbs. It is widely applied in the food field; it is used to enhance the fragrance and flavor in cooking, and in the beverage and the chewing gum industries (de Carvalho and da Fonseca, 2006). In many studies, L-carvone has been reported as an antimicrobial agent for foodborne pathogenic microorganisms (Friedman et al., 2002; Porfírio et al., 2017); however, there is still limited information about the relationship between spoilage bacteria and QSIs. Therefore, our study involves the characterization of the L-carvone-mediated inhibition of the QS activity of the biosensor strain *Chromobacterium violaceum* CV026, and subsequently, the determination of the effect of L-carvone on virulence factor and biofilm production in the spoilage bacterium *H. alvei*. Additionally, we further investigated the underlying mechanism of L-carvone as a potential QSI in *H. alvei*, by using the *in silico* analysis and RT-qPCR techniques. In this regard, the study has provided new information about the application of L-carvone as potential QSI and reference values for the effective control of spoilage bacteria.

## Materials and Methods

### Reagents, Bacterial Strains, and Growth Conditions

L-Carvone (≥99% purity) and AHL standards including C_4_-HSL, C_6_-HSL, C_8_-HSL, C_10_-HSL, C_12_-HSL, and C_14_-HSL were obtained from Sigma-Aldrich (United States). The molecular biology reagents were purchased from Thermo Fisher Scientific (Shanghai, China). Other chemical reagents used in this study were of analytical grade, except for methanol (Chromatographic grade). The bacterial strains used in this study were *C. violaceum* CV026 and *H. alvei* Ha-01, as an AHL-reporter organism and a test strain, respectively. *C. violaceum* CV026 was provided by Dr. Yang (Xinjiang Shihezi University, Xinjiang, China) and *H. alvei* (ATCC 13337) Ha-01 was originally isolated and identified from putrid turbot by our group. *C. violaceum* CV026 was a mini-Tn5 mutant derived from *C. violaceum* ATCC 31532; it was kanamycin-resistant. It could respond only when exogenous AHLs were present, after which it produced the characteristic violet pigment, violacein. Both the strains were overnight cultured in Luria-Bertani (LB) broth (Qingdao Hopebio Co., Ltd., China), at 28°C and 160 rpm; however, the LB broth culture medium for CV026 required 20 μg/mL kanamycin.

### Antibacterial Assay

#### Determination of the Minimum Inhibitory Concentration (MIC) of L-Carvone

The MIC of L-carvone against the selected bacteria was determined using the Oxford cup assay method, as described by Diao et al. (2014). Overnight-cultured (OD_600_ = 0.5, 250 μL) *C. violaceum* CV026 or *H. alvei* was inoculated in LB nutrient agar (25 mL) and poured into a plate that accommodated two autoclaved Oxford cups, which were removed when the agar solidified. Two hundred microliters of L-carvone (diluted to 2.0, 1.0, 0.5, 0.25, 0.125, and 0.0625 μL/mL using sterile water) were added to the wells, while sterile water served as the control. The plates were incubated at 28°C for 36 h and the bacterial growth states were observed. The minimum concentration at which there was no visible growth was defined as the MIC. Then, sub-MICs were selected for the further experiments using the above strains.

### Determination of QSI Activity

#### Violacein Inhibitory Activity

The violacein inhibitory activity was determined by adopting the method described by Ia et al. (2012), with slight modifications. Overnight-cultured *C. violaceum* CV026 (250 μL) was inoculated in LB nutrient agar (25 mL) containing 10 μL of exogenous AHLs (C_6_-HSL, 2 mg/mL). Afterwards, 200 μL of L-carvone at the sub-MICs was added to each well (diameter, 6 mm) on the plates, while 200 μL of sterilized water was used as the negative control. The plates were incubated at 28°C for 24 h, and the bacterial growth status was observed. Once no violet pigment was produced around the well, the violacein inhibitory activity was determined.

#### Quantitative Analysis of Violacein Production

Violacein produced by *C. violaceum* CV026 exposed to different concentrations (0.5, 0.25, 0.125, and 0.0625 μL/mL) of L-carvone was quantified as previously described by Choo et al. (2006). Different concentrations of L-carvone (described above) were mixed in 10 mL of LB broth containing 20 μg/mL C_6_-HSL, along with *C. violaceum* CV026 overnight cultures, and incubated at 28°C for 48 h with shaking (160 rpm). At the same time, a similar experiment without C_6_-HSL was performed, and the OD_595_ was measured to determine the effect of the above concentrations of L-carvone on the growth of the CV026.

The violacein pigment was extracted according to the method described by Kumar et al. (2015) with modifications. The cultures in each treatment group were vortexed, and 300 μL of these mixed cultures were taken in 1.5-mL tubes (Eppendorf). They were lysed (for 15 s) using 10% sodium dodecyl sulfate (SDS, 150 μL) at room temperature, and then, extracted (for 5 s) using butyl alcohol (600 μL). Finally, this solution was centrifuged (9,000 *g* for 5 min); violacein was contained in the organic layer. Then, the OD_595_ of each supernatant was measured in a 96-well microtiter plate.

### Assay for Biofilm Formation

The 1.5-mL Eppendorf tubes (polypropylene material) were autoclaved, and the *H. alvei* overnight cultures (100 μL) were inoculated in 1 mL of LB broth containing various concentrations (0.5, 0.25, 0.125, and 0.0625 μL/mL) of L-carvone. Sterile water or 20 μg/mL C_6_-HSL was used as the negative control or positive control (absence of L-carvone), respectively. The tubes were statically incubated at 28°C for 48 h. Then, the determination of biofilm was performed as described previously (Rode et al., 2007), with minor modifications. The cultures were discarded, and each tube was rinsed thrice with sterile water. The tubes were then naturally dried for 40 min and stained with 1 mL of 0.1% crystal violet (w/v) for 15 min at room temperature. After washing with sterile water, the biofilms were extracted using 33% acetic acid. The biofilm solutions were then transferred to a clean 96-well plate, and the OD_595_ values were measured using microplate photometers (Bio-Rad, United States).

### Visualization of Biofilms by CLSM and SEM

To pre-form the biofilms, pieces of zinc (6 mm × 6 mm × 0.2 mm) were polished and immersed in LB broth containing sub-MICs of L-carvone or 20 μg/mL of C_6_-HSL in 90-mm sterile plates (Thermo, United States). Overnight cultures of *H. alvei* (OD_600_ = 0.5, 100 μL) were inoculated in these plates and then statically incubated. After cultivation (at 28°C for 48 h), a piece of zinc (with an adhered biofilm) was transferred to a clean sterile plate and washed thrice with sterile phosphate buffer saline (PBS, pH 7.4) to remove the planktonic cells. For visualization by confocal laser scanning microscopy (CLSM), this zinc piece was stained with 0.01% (w/v) acridine orange (AO, dissolved in PBS) for 15 min in the dark. Then, the excessive AO was removed by washing with PBS, followed by fixing with antifade mounting medium Fluoromount-G^TM^ (Yeasen, China) for 15 min under the same conditions. Finally, the samples were observed by CLSM (Leica SP5, German) (emission: 525 nm, excitation: 488 nm). For visualization analysis by scanning electron microscopy (SEM), the zinc piece was soaked in 2.5% glutaraldehyde (v/v) at 4°C for 5 h, dehydrated in graded ethanol (15 min for each grade). Subsequently, the SEM sample was obtained after drying with sterile air.

### Swimming and Swarming Motility Assay

Motility experiments were performed on swimming (1% [w/v] tryptone, 0.5% [w/v] NaCl, and 0.3% [w/v] agar) or swarming (1% [w/v] peptone, 0.5% [w/v] NaCl, 0.5% [w/v] D-fructose, and 0.6% [w/v] agar) agar plates, as previously described (de la Fuente-Núñez et al., 2012), but with some modifications. These agar plates were supplemented with different concentrations (0.5, 0.25, 0.125, and 0.0625 μL/mL) of the L-carvone, before the agar solidified. Then, 5 μL of *H. alvei* overnight cultures (OD_600_ = 0.5) was inoculated at the center of the solidified plates, and the plate was incubated statically at 28°C for 48 h. The motility of *H. alvei* was evaluated by measuring the diameter of the swimming and swarming colonies. Plates supplemented with sterile water or 20 μg/mL C_6_-HSL were used as a negative or positive control, respectively. At least three independent experiments for motility assays were performed.

### AHL Analysis by GC-MS

#### AHLs Extraction

Dilutions (1/100) of *H. alvei* overnight cultures were incubated in LB broth (200 mL) in the presence of L-carvone (0.5, 0.25, 0.125, and 0.0625 μL/mL) for 24 h at 28°C in an Erlenmeyer flask. Bacterial cells were removed by centrifugation (9,000 *g* for 15 min). The supernatants were extracted using ethyl acetate supplemented with 0.1% acetic acid thrice, and then, the organic phases were evaporated using a rotary evaporator. The residues were re-dissolved in methanol (1 mL) and filtered through a 0.22-μm membrane (FilterBio, China) for GC-MS detection. For comparison, LB broth in the absence of L-carvone was used. C_14_-HSL, as an internal standard, was added to each of the AHL samples at a concentration of 5 μg/mL.

#### GC-MS Detection

The AHL samples of *H. alvei* were further analyzed by GC-MS (7890N/5975, Agilent, United States), according to the method described by Zhu et al. (2016). A HP-5 MS capillary column (30 m length × 0.25 mm internal diameter × 0.25 μm film thickness) was used for the chromatographic separation of the AHLs. The injection volume was 1 μL, using a slit ratio of 50:1. The injector temperature was maintained at 200°C and the oven temperature was automated from 150 to 220°C at a rate of 10°C/min, followed by a 5°C/min increase to 250°C, and from 250 to 252.5°C at 0.5°C/min, with helium as the carrier gas, at a flow rate of 1 mL/min. The mass spectrometry conditions were as follows: the electron ionization source was set to 70 eV, the MS Quad temperature was 150°C, the emission current was 500 μA, the MS Source temperature was 230°C. Data were acquired using the full-scan mode (m/z 35–800) and selected ion monitoring (SIM) mode (m/z 143).

### RT-qPCR Analysis

*Hafnia alvei* was cultured in LB broth with sub-MICs of L-carvone at 28°C for growth until the logarithmic phase. *H. alvei* cultured without L-carvone was used as a negative control, while *H. alvei* cultured with C_6_-HSL was used as a positive control. Total RNA was isolated from *H. alvei* using TRIzol Reagent (Thermo Scientific, United States), according to the manufacturer’s guidelines. The quality of the isolated RNA was checked using standard agarose gel electrophoresis. The single-stranded cDNA was prepared in accordance with the protocol of RevertAid First Strand cDNA Synthesis Kit & DNase I (Thermo Scientific, United States), as stated by the manufacturer. The RT-qPCR experiment was performed by using BIO-RAD CFX Connect^TM^ Real-Time PCR test system (BIO-RAD, United States) and Power SYBR^®^ Green PCR Master Mix (Applied Biosystems, United States). The sequences of primers are listed in Table 1. The housekeeping gene *16S rRNA* was used as an internal reference. The conditions for RT-qPCR were as follows: initial denaturation at 95°C for 3 min, and 95°C for 10 s, 55°C for 20 s for annealing, 72°C for 20 s for extension, and 75°C for 5 s for collecting the fluorescence signal; 40 cycles were run. The melt curve was established in the range of 65–95°C. The relative expression of the objective genes was calculated by using the 2^-ΔΔCT^ method, as previously described by Livak and Schmittgen (2001).

**Table 1:** List of target genes and their respective primers used for RT-qPCR analysis.

Target gene	Primer type	Sequence (5′→3′)	Amplicon size (bp)
*16S rRNA*	Forward	GTCTGCAACTCGACTCCATGA	121
	Reverse	CTTTTGCAACCCACTCCCATG	
*halI*	Forward	CGGCTTGATTCCACTTCACC	132
	Reverse	GGGTCTGTATGAAGGGCAGT	
*halR*	Forward	CGGCTATACTTTCGTCCTGC	179
	Reverse	CTTCCGAAACTGACTGCACG	


*The lux-type genes in H. alvei were halI and halR, respectively.*

### *In silico* Analysis

The lux-type protein sequences of *H. alvei* were HalI (acyl-homoserine-lactone synthase, AAP30849.1) and HalR (transcriptional regulators, AAP30848.1) and downloaded from the NCBI^1^. The models of these proteins were built and assessed the online tools SWISS-MODEL^2^ (Bertoni et al., 2017; Bienert et al., 2017; Waterhouse et al., 2018) for docking studies. The water molecules associated with the protein model were removed and the missing hydrogen atoms were supplemented using Clean Protein module of Discovery Studio (DS). The 3D structures of the ligands including L-carvone, halogenated furanone C30 (a known QSI), and C_6_-HSL were downloaded from the ZINC 12 database^3^ and optimized in DS to obtain their possible lowest-energy conformations. The binding spheres that covered the active site residues were also obtained with DS, using the Define and Edit Binding Site module. Finally, docking of the ligands was subsequently performed using the Libdock algorithm.

### Statistical Analysis

Each experiment was performed in triplicate, and the data were presented as the mean values ± SD. The data were analyzed by one-way analysis of variance (ANOVA) along with Tukey test correction using the software SPSS Statistics 20.0. Graphs were constructed using Origin Pro 9.0. Differences with a *p*-value < 0.05 were considered significant.

## Results

### Minimum Inhibitory Concentration (MIC) of L-Carvone

The MIC of L-carvone, with concentrations ranging from 0.0625 to 2 μL/mL, was estimated by the Oxford cup method. It was observed that the MIC of L-carvone for *C. violaceum* CV026 was 1.0 μL/mL; L-carvone did not influence the growth of *H. alvei* at the same concentration. Therefore, the sub-MICs (0.5, 0.25, 0.125, and 0.0625 μL/mL) were selected for further experiments in this study.

### Effect of L-Carvone on Violacein Production in *C. violaceum* CV026

**FIGURE 1:**
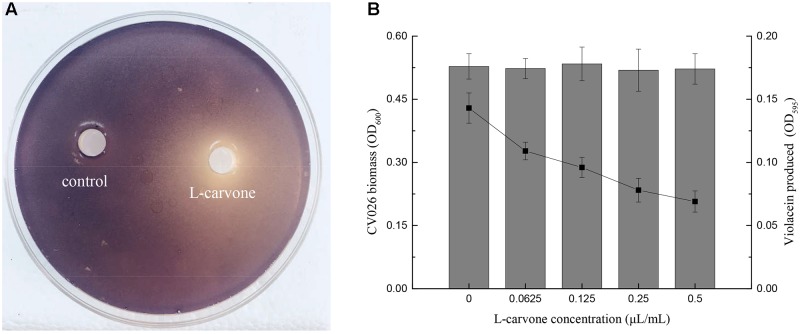
Violacein inhibitory activity of L-carvone. **(A)** Inhibitory activity of L-carvone on the violacein production of CV026. **(B)** Quantitative analysis of CV026 biomass (columns) and violacein production (lines) at sub-inhibitory concentrations of L-carvone.

To determine whether L-carvone at the sub-MICs inhibited violacein production in CV026, two assays were performed. Figure 1A shows that a clear inhibitory zone was observed around the well on the purple pigment plate due to L-carvone; however, the control, sterile water, did not inhibit pigment production. Furthermore, the quantitative results of violacein production were obtained. L-Carvone showed a dose-dependent QSI activity and did not significantly inhibit bacterial growth at the sub-MICs (Figure 1B). The minimum violacein production rate (OD treatment group/OD control group) was only 48.25% at a 0.5 μL/mL concentration of L-carvone.

### Effect of L-Carvone on Biofilm Formation in *H. alvei*

The results of biofilm formation after treatment with different concentrations of L-carvone are presented in Table 2. A minimum biofilm inhibition of 13.43% was observed when *H. alvei* was cultured with L-carvone at 0.0625 μL/mL; a maximum biofilm inhibition of 52.41% was observed at am L-carvone concentration of 0.5 μL/mL. In contrast, biofilm formation in the C_6_-HSL-treated group was visibly higher than that in the control group, which proves that the biofilm formation of *H. alvei* is positively regulated by the AHL-based QS system.

**Table 2:** Inhibitory activity of L-carvone on biofilm formation by *H. alvei* (mean ± standard deviation).

Additive	Concentration	Biofilm formation^a^	Inhibitory rate (%)^b^
C_6_-HSL	20 μg/mL	0.951 ± 0.004^c^	–
Control	0 μL/mL	0.685 ± 0.004^d^	–
L-carvone	0.0625 μL/mL	0.593 ± 0.007^e^	13.43
L-carvone	0.125 μL/mL	0.532 ± 0.010^e^	15.30
L-carvone	0.25 μL/mL	0.478 ± 0.003^f^	30.32
L-carvone	0.5 μL/mL	0.326 ± 0.005^g^	52.41


*^a^Expressed as OD_595_ after incubation with crystal violet. ^b^The inhibitory rate = (OD control group - OD treated group)/OD control group. ^c-g^Significantly different means (P < 0.05).*

**FIGURE 2:**
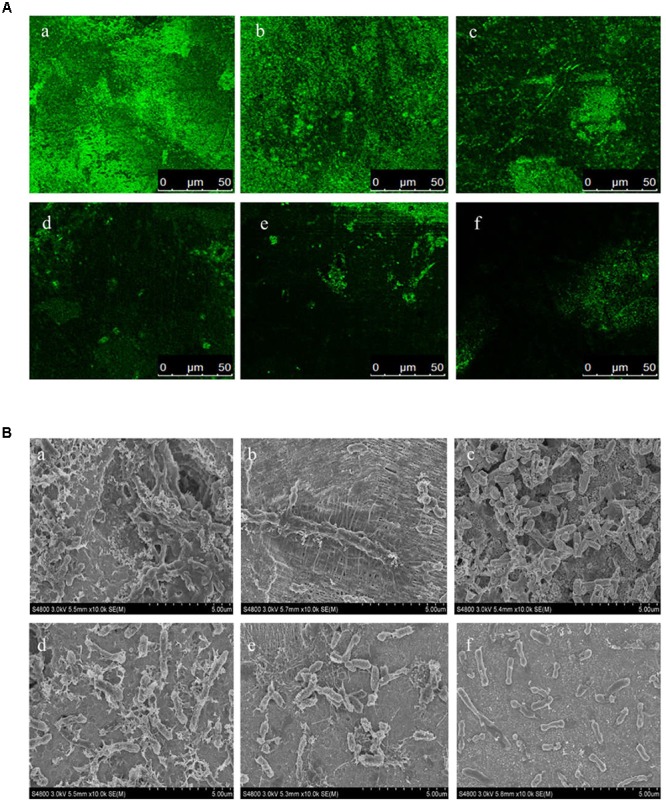
Confocal laser scanning microscopy (CLSM) images **(A)** and SEM images **(B)** of the biofilm states of the *H. alvei* strain on zinc surfaces after different treatments. **(a)** 20 μg/mL C_6_-HSL, **(b)** control, **(c–f)** L-carvone treatments at concentrations of 0.0625, 0.125, 0.25, and 0.5 μL/mL.

In this study, the biofilm states of the *H. alvei* strain in the presence of various concentrations of L-carvone were also observed by CLSM and SEM. The CLSM images showed thick and dense biofilms after C_6_-HSL treatment, compared with the control group (Figure 2A), whereas L-carvone treatment significantly removed the microbes attached to the zinc surface (Figure 2A). The SEM images displayed similar results and showed a major disruption to the biofilm architecture as well as the reduction of the biofilm matrix (Figure 2B).

### Effect of L-Carvone on Swimming and Swarming Motility of *H. alvei*

**FIGURE 3:**
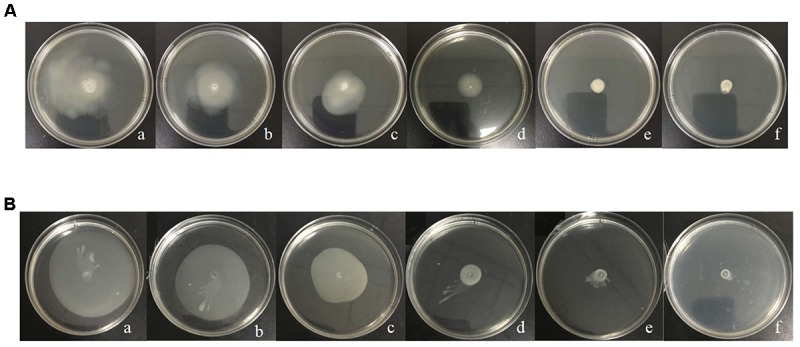
Analysis of the inhibition of the motility of *H. alvei* by L-carvone. Swimming **(A)** and swarming **(B)** agar plates were incubated at 28°C and monitored after 48 h. **(a)** 20 μg/mL C6-HSL, **(b)** control, **(c–f)** 0.0625, 0.125, 0.25, and 0.50 μL/mL L-carvone.

The migration distance of *H. alvei* grown on swimming and swarming agar plates at 28°C for 48 h is shown in Figure 3. The treatment of *H. alvei* with sub-MICs of L-carvone reduced the swimming motility significantly; the level of swimming motility inhibition due to L-carvone (0.0625–0.5 μL/mL) was 12.43–61.49%, as depicted in Supplementary Table S1. Similarly, swarming migration of *H. alvei* was also impaired considerably (23.29–74.94%) after treatment with L-carvone (Supplementary Table S1). However, the treatment of *H. alvei* with C_6_-HSL promoted its motility.

### Effect of L-Carvone on AHL Production in *H. alvei*

To investigate the effect of L-carvone on AHL production of test strain, the AHLs in the ethyl acetate crude extract of *H. alvei* were analyzed using GC-MS. After the AHL standards were separated individually, and their retention times were identified (Supplementary Figure S1A), we calculated the relative quantity of AHLs in the crude extracts based on the ratio of the peak area of the samples to that of the internal standard (C_14_-HSL). The AHL types observed in the *H. alvei* crude extracts were C_6_-HSL and C_8_-HSL, at concentrations of 2.16 ± 0.06 and 2.27 ± 0.12 μg/mL, respectively. Treatment with L-carvone significantly reduced the AHL production (Supplementary Figure S1C); when treated with 0.5 μL/mL L-carvone, the minimal concentrations of C_6_-HSL and C_8_-HSL decreased to 0.16 ± 0.09 and 0.97 ± 0.04 μg/mL, respectively (Supplementary Figure S1B).

### RT-qPCR

**FIGURE 4:**
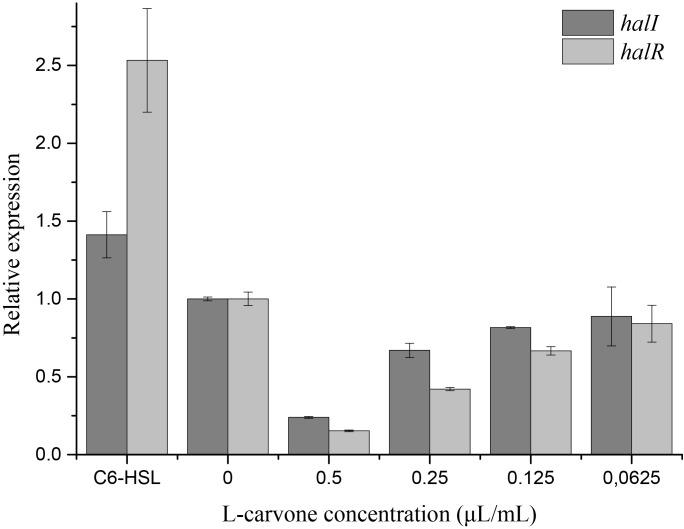
Effect of L-carvone on the expression of QS-regulated genes of *H. alvei*. The lux-type genes of the QS system in *H. alvei* were *halI* and *halR*, respectively. The untreated group was used as a negative control, and the group treated with C_6_-HSL was used as a positive control. The relative expression was considered as 1.

The RT-qPCR experiments were performed to understand the effect of L-carvone on the expression level of QS-regulated genes in *H. alvei*. The selected genes were lux-type genes, named *halI* and *halR*, respectively. Because in this QS system, the *halI* gene regulated AHL biosynthesis by encoding HalI (the AHL synthase), the *halR* gene responded to the corresponding AHL by encoding HalR (the transcriptional regulator), and further regulated the transcription of the downstream genes. The results obtained in this study show that L-carvone could selectively affect the QS system by significantly downregulating the relative expression levels of *halI* and *halR* (Figure 4). C_6_-HSL, which was used as the positive control, could significantly upregulate the expression of the selected genes. Melt and amplification curves of the genes were established in Supplementary Figure S3.

### Homology Modeling and Model Assessment

At present, the three-dimensional (3D) structures of the HalI and HalR proteins have not yet been analyzed; therefore, homology modeling, based on the online tool SWISS-MODEL, was utilized to solve this problem. Modeling templates were matched using the amino acid sequences of HalI (acyl-homoserine-lactone synthase, AAP30849.1) and HalR (transcriptional regulators, AAP30848.1); the top 50 templates of each protein were obtained. The three best models of HalI and HalI proteins are listed in Supplementary Table S2, based on sequence similarities and the GMQE scores.

Model qualities were assessed by using QMEAN, which is a composite estimator and provides both global and local absolute quality estimates for models (Benkert et al., 2011). QMEAN *Z*-Scores of around zero are an indication of a high quality for a model; however, scores of -4.0 or lower indicated a low quality. Therefore, the results in Supplementary Figures S2A–D show that the best models for HalI and HalR proteins were the 1k4j.1.A (score of -1.85) and 5l07.1.B (score of -1.37) models, respectively; they were able to efficiently predict the 3D structures of Lux-type proteins in *H. alvei*.

### *In silico* Analysis

*In silico* analysis studies of L-carvone provide an insight into the binding affinity of this potential QSI, with the model of HalI and HalR protein. For the QS transcriptional receptor HalR protein, the halogenated furanone C30 and C_6_-HSL were docked as the control ligands. As shown in Supplementary Table S3, L-carvone docked with the active site of the HalI protein of *H. alvei*, with a LibDock score of 71.0676. Moreover, L-carvone (LibDock score of 66.7963) showed a better affinity toward HalR than the standard QSI, halogenated furanone C30 (LibDock score of 52.7221). However, both the ligands were observed to have a lower affinity toward HalR than the natural ligand C_6_-HSL (LibDock score of 84.7765). Figure 4 depicts the possible mechanism of the action of L-carvone in attenuating QS-regulated virulence factor and biofilm production in *H. alvei*.

## Discussion

There is increasing evidence that plant essential oils can act as potential QSIs, to reduce QS-mediated production of virulence factors and biofilms in microorganisms, and provide a new insight into controlling microbial communities (Zhang et al., 2018). Our data support this notion, revealing a potential QSI, L-carvone (the main component of spearmint essential oil), which interferes with violacein expression in *C. violaceum* CV026 and enters *H. alvei*, reducing its motility, biofilm formation, and expression of QS-related genes.

In this study, originally, sub-MICs of L-carvone were tested for their QSI activity using the CV026 strain (Figure 1). The biosensor strain CV026 can only respond to exogenous short-chain AHLs through the cytoplasmic transcription factor *CviR* (a LuxR homolog), which activates the expression of violacein in combination with the AHLs (McClean et al., 1997). Many studies have revealed that the reduction of violacein production without the growth of CV026 being affected is considered a direct evidence for the interference of the QS system (Venkadesaperumal et al., 2016; Liu et al., 2017). Based on the above evidences, this work further explored the QS interference activity of L-carvone on *H. alvei*, since the QS-mediated production of virulence factors and biofilms plays a key role in the growth of this spoilage organism (Hou et al., 2017b).

The bacterial cells in biofilms are more resistant to antiseptics and food processing conditions; this is likely to cause serious food safety issues (Bai and Rai, 2011). Consequently, studies on preventing biofilm formation are garnering special interest. Previous studies have indicated that the effects of L-carvone on biofilms of Gram-positive and Gram-negative bacteria are possibly different. Soumya et al. (2011) reported that the sub-MICs of L-carvone could reduce biofilm formation in *Pseudomonas aeruginosa* as a natural QS-inhibitory compound. However, in case of Gram-positive bacteria, the study by Leonard et al. (2010) indicated that carvone could increase biofilm production in *Listeria monocytogenes*, rather than inhibiting its production. Interestingly, Oliveroverbel et al. (2014) also showed that carvone could inhibit violacein and pyocyanin production in *C. violaceum* and *P. aeruginosa*, respectively, by interfering with their QS systems, and found that this inhibition was produced by its levorotary analog. Herein, for the first time, we have reported that at sub-MICs, L-carvone, a potential natural QSI, could significantly reduce biofilm formation by *H. alvei* at 28°C on polypropylene and zinc surfaces (Table 2 and Figure 2). This result was similar to those of the reports of Soumya and Oliveroverbel. A maximum inhibition of 52.41% was observed using a microplate photometer. Furthermore, *in situ* analysis of the biofilm matrix performed using SEM and LCSM was able to provide further information on the structure of the formed biofilms following different treatments (Azeredo et al., 2017). As reported by Gross (2017), biofilms were seen as ‘Microbial cities’ that included both infrastructure (generally embedded in polysaccharide matrixes) and social communication. CLSM and SEM images in our study clearly displayed a major disruption to this infrastructure and the reduction to the biofilm matrix (Figure 2). These results were similar to those of the study by Zhou et al. (2018), who found the Hordenine (a sprouting barley extract) could act as a novel QSI and inhibit biofilm formation in *P. aeruginosa*.

Quorum sensing-regulated flagellar-dependent motility (like swimming and swarming) is closely associated with biofilm formation. In addition, this motility (QS-regulated flagellar-dependent motility) is considered as a virulence factor because of its fundamental role in adhesion, colonization, and virulence expression of pathogens (Atkinson et al., 2006; Bluskadosh et al., 2013). Therefore, a decrease in motility would likely control the biofilm formation of *H. alvei* and weaken its infection ability. In the present study, treatments with L-carvone dose dependently inhibited the migration capacity of *H. alvei*. An L-carvone concentration of 0.5 μL/mL showed that the maximum inhibition levels of swimming and swarming motility were 61.49 and 74.94%, respectively. These results are consistent with those from an earlier study by Hou et al. (2017a), who demonstrated a significant inhibition of motility in *H. alvei* by the food additive dihydrocoumarin.

Due to the essential role of AHLs on the QS system, the effects of L-carvone treatment were characterized using GC-MS, and major changes in the AHL production by the *H. alvei* strain were observed. GC-MS, with the electron ionization mode, is a powerful tool for the rapid, easy, and selective determination of the AHL levels (Cataldi et al., 2004). The results indicated that L-carvone was able to significantly inhibit the production of both the primary AHLs (C_6_-HSL and C_8_-HSL) in *H. alvei*, especially reducing the C_6_-HSL production from 2.16 to 0.16 μg/mL. Similarly, Luciardi et al. (2016) found that volatiles from food and medicinal plants could interfere with QS-mediated virulence expression in *P. aeruginosa* by reducing the biosynthesis of AHLs.

**FIGURE 5:**
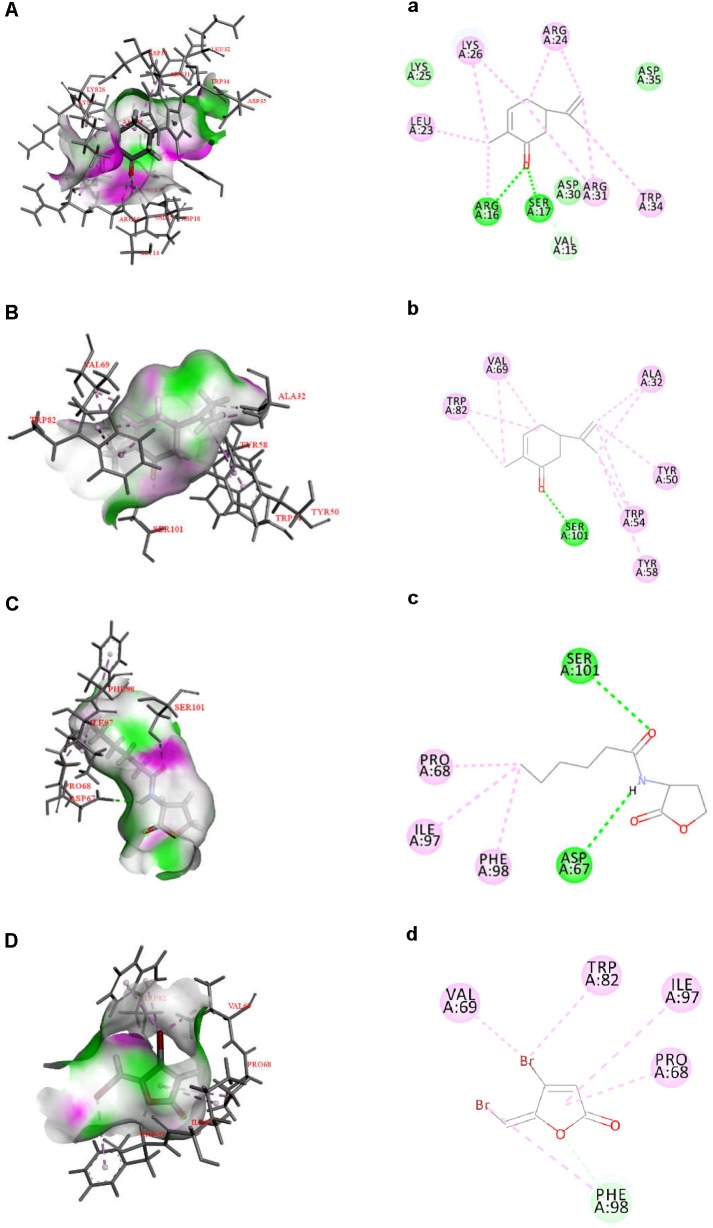
Docked interactions of the native ligand C_6_-HSL and compounds L-carvone as well as halogenated furanone C30 with the HalI and HalR protein model are shown as 2D diagrams **(A–D)** and 3D diagrams **(a–d)**. The key amino acids of the proteins are shown as thin sticks, ligands are shown as thick sticks, hydrogen bonds are shown as green lines, and hydrophobic forces are shown using a light violet color. Receptor surfaces were displayed as H bonds.

Quorum sensing in Gram-negative bacteria is predominantly controlled by LuxI/R-type proteins, which regulate the production of AHLs, expression of virulence factors, and formation of biofilms (Fuqua et al., 1994). To investigate the inhibitory mechanism of L-carvone on the QS system of *H. alvei*, relevant protein-molecular interactions were firstly evaluated by *in silico* analysis. According to the *in silico* results, we noticed high LibDock scores of the docking of L-carvone with the HalI (LuxI-type protein) and HalR (the LuxR-type protein) of *H. alvei* (Supplementary Table S3). In HalI, L-carvone was well embedded into a cavity in the vicinity of the active site, the key residues of which included ARG16, SER17, VAL15, ARG31, TRP34, ARG24, LYS26, and LEU23. Simultaneously, L-carvone formed three hydrogen bonds with ARG16, SER17, and VAL15 and showed a hydrophobic behavior with the other residues, as shown in Figures 5A,a. In HalR, L-carvone formed one hydrogen bond with SER101 and interacted with other residues (TRP82, VAL69, ALA32, TYR50, TRP54, TYR58, and SER101) via the hydrophobic effect (Figures 5B,b). C_6_-HSL, as a positive control, formed two hydrogen bonds with SER101 and ASP67 (Figures 5C,c). However, the standard QSI, halogenated furanone C30, as a negative control, did not form any hydrogen bonds with HalR (Figures 5D,d).

The hydrogen-bonding interactions are considered to play a major role in the process where ligands dock with the LuxR-type receptor (Gerdt et al., 2015). In our study, L-carvone showed a better *in silico* affinity toward HalR than the halogenated furanone C30, because of a higher LibDock score and additional hydrogen bonds. L-carvone and C_6_-HSL can form hydrogen bonds with the HalR protein at a common site, SER101, indicating a possible competitive action between them. Combined with the GC-MS results, these data confirm that the inhibitory mechanism of L-carvone on the QS system of *H. alvei* might involve the interaction of L-carvone with the HalI protein and subsequent interference of AHL biosynthesis in *H. alvei*. In addition, we also characterized the effects of L-carvone treatment using transcriptomics, and observed that the *halI* and *halR* genes were significantly downregulated in *H. alvei*, similar to the results reported in a previous research study (Zhou et al., 2018). The RT-qPCR results were consistent with those of the *in silico* analysis, which enhanced the credibility of the QS inhibitory mechanism of L-carvone.

## Conclusion

In summary, the present study demonstrates that L-carvone had a significant inhibitory activity on the QS system by reducing the AHL-mediated production of virulence factors and biofilm formation in *H. alvei*. More specifically, L-carvone combined with the AHL synthase HalI via hydrogen bonds, which led to the disruption of AHL biosynthesis. Understanding the roles and functions of QS in food ecosystems can help in preventing the colonization of food surfaces, toxin formation, and proliferation of food-related bacteria. Therefore, L-carvone, with a QS inhibitory activity, is a promising agent for controlling foodborne pathogens and improving food safety.

## Author Contributions

TL and YM contributed to the conception of the study. YM performed the data analyses and wrote the manuscript. BH and XS contributed significantly to analysis and manuscript preparation. JL helped to perform the analysis with constructive discussions. All authors contributed to manuscript revision, read and approved the submitted version.

## Conflict of Interest Statement

The authors declare that the research was conducted in the absence of any commercial or financial relationships that could be construed as a potential conflict of interest.

## Footnotes

1 https://www.ncbi.nlm.nih.gov/

2 https://www.swissmodel.expasy.org/

3 http://zinc.docking.org/
