# Supplementary material for: Reducing Quorum Sensing-Mediated Virulence Factor Expression and Biofilm Formation in Hafnia alvei by Using the Potential Quorum Sensing Inhibitor L-Carvone
Source: Front Microbiol. 2019 Jan 9;9:3324. doi: 10.3389/fmicb.2018.03324 (PMC6334668; doi:10.3389/fmicb.2018.03324)
Supplement: Supplementary file 1 [file Data_Sheet_1.doc]

**Journal name**: Frontiers in Microbiology

**Manuscript Title**:

**Reducing Quorum Sensing-Mediated Virulence Factor Expression and Biofilm Formation in *Hafnia alvei* by Using the Potential Quorum Sensing Inhibitor L-Carvone**

**Authors**: Tingting Li^1^, Yongchao Mei^2,3^, Binbin He^2,3^, Xiaojia Sun^2,3^, Jianrong Li^2,3*^

^1^ Key Laboratory of Biotechnology and Bioresources Utilization (Dalian Minzu University), Ministry of Education, Dalian, China

^2^ College of Food Science and Technology, Bohai University, Jinzhou, China

^3^ National & Local Joint Engineering Research Center of Storage, Processing and Safety Control Technology for Fresh Agricultural and Aquatic Products, Jinzhou, China

*Author for correspondence. Tel/Fax: +86-416-3400008

E-mail address:

Tingting Li: tingting780612@163.com

Yongchao Mei: [myc9406@163.com](mailto:myc9406@163.com)

Binbin He: 15241629772@163.com

Xiaojia Sun: sunxj27@163.com

Jianrong Li*: [lijr6491@163.com](mailto:lijr6491@163.com)


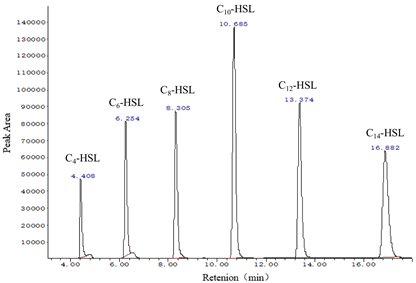

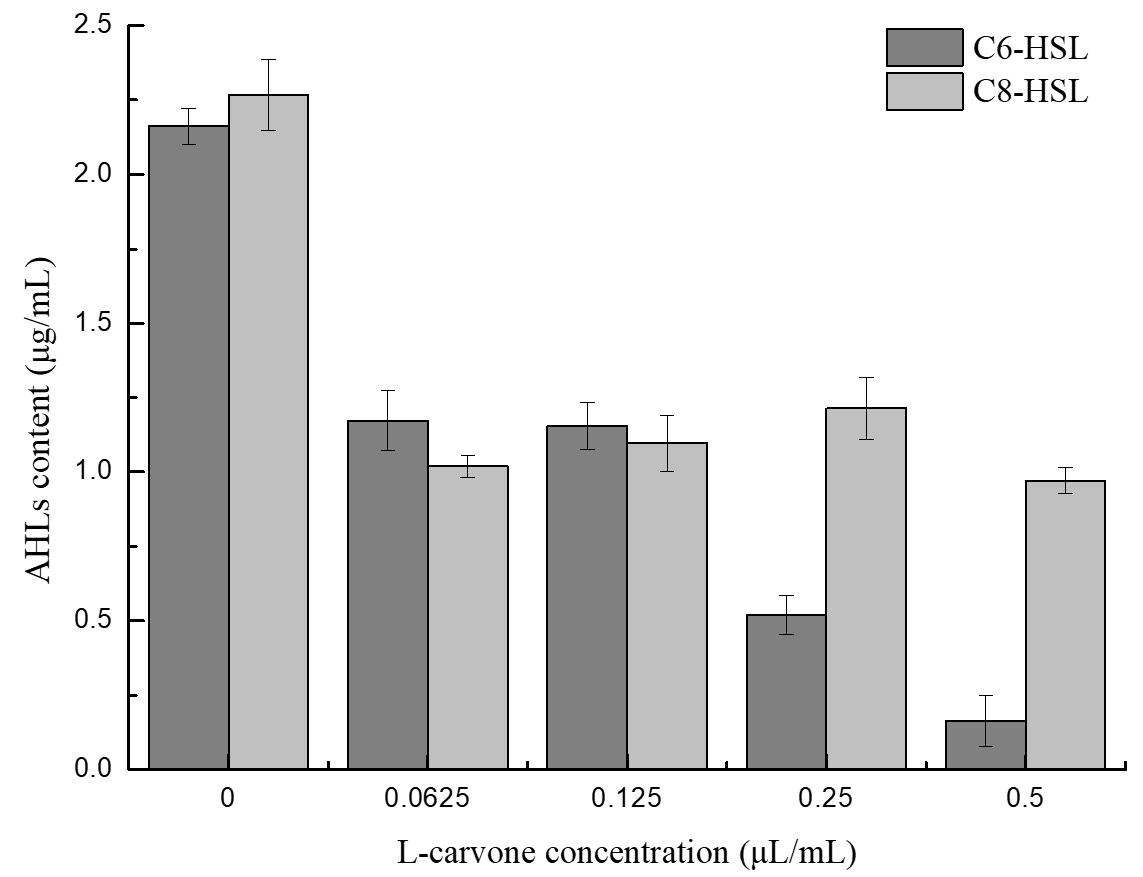


1. (B)

**
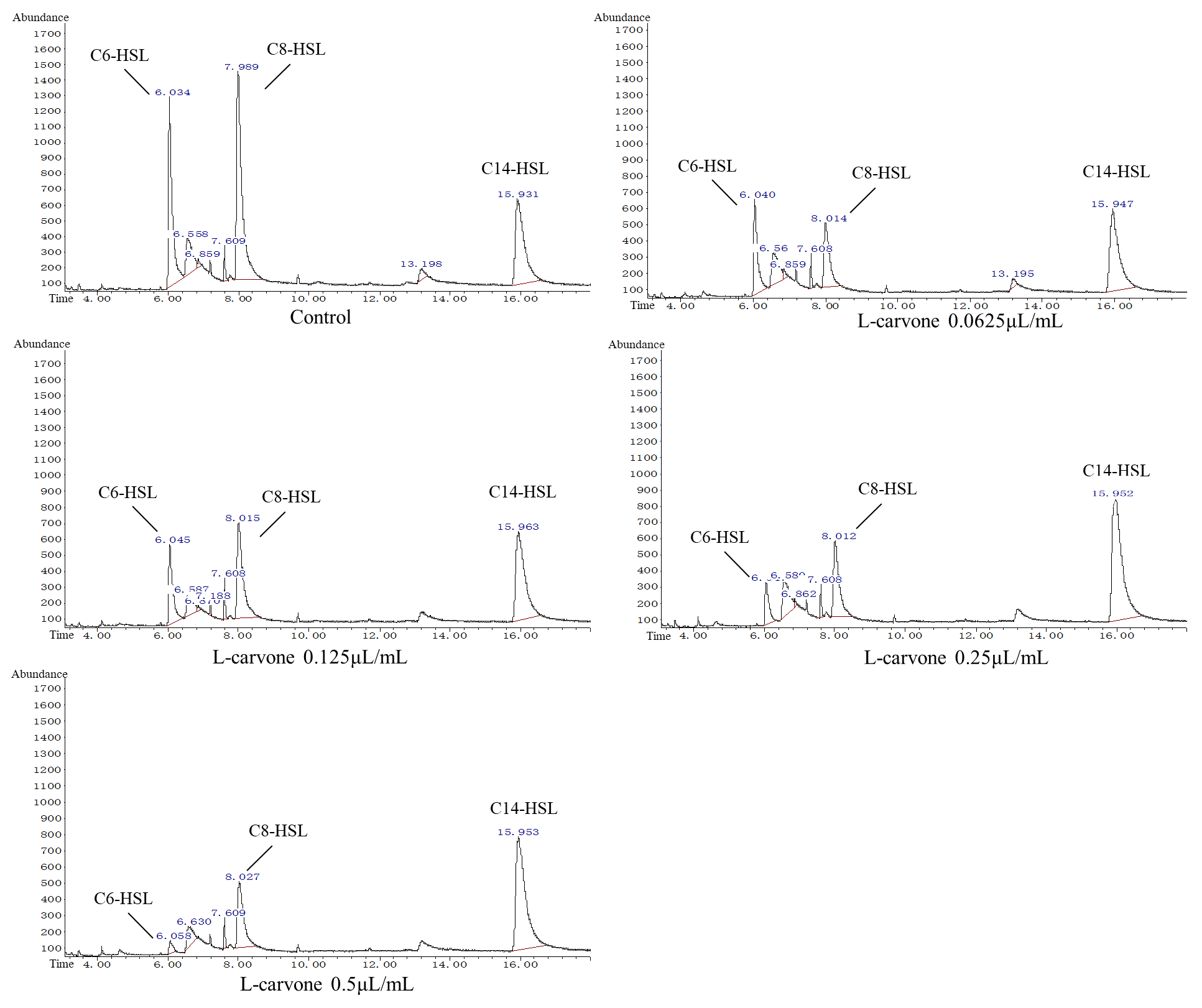
**

(C)

**Figure S1. The results of GC-MS analysis**

1. The chromatogram of AHL standards; (B) The effects of L-carvone on AHL production in *H. alvei*; (C) The chromatograms of AHL by different treatments

**
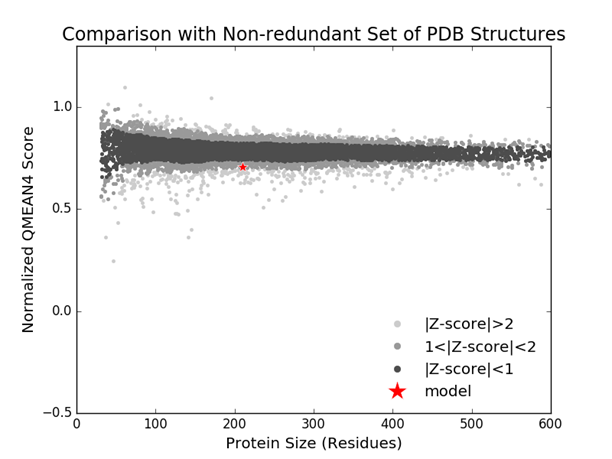

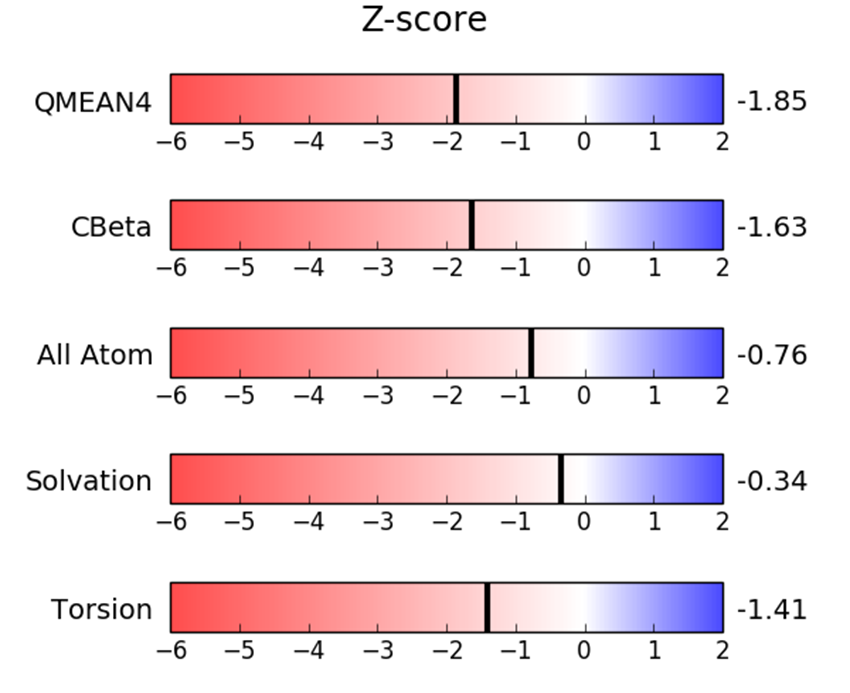
**

1. **(B)**

**
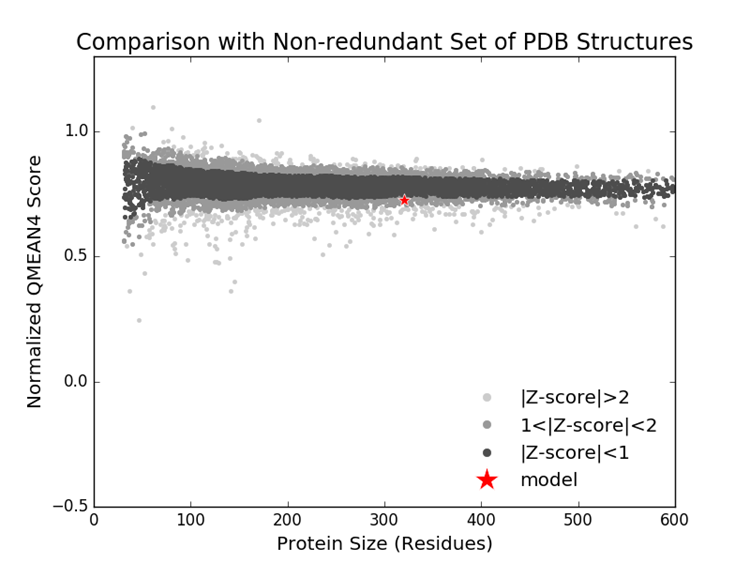

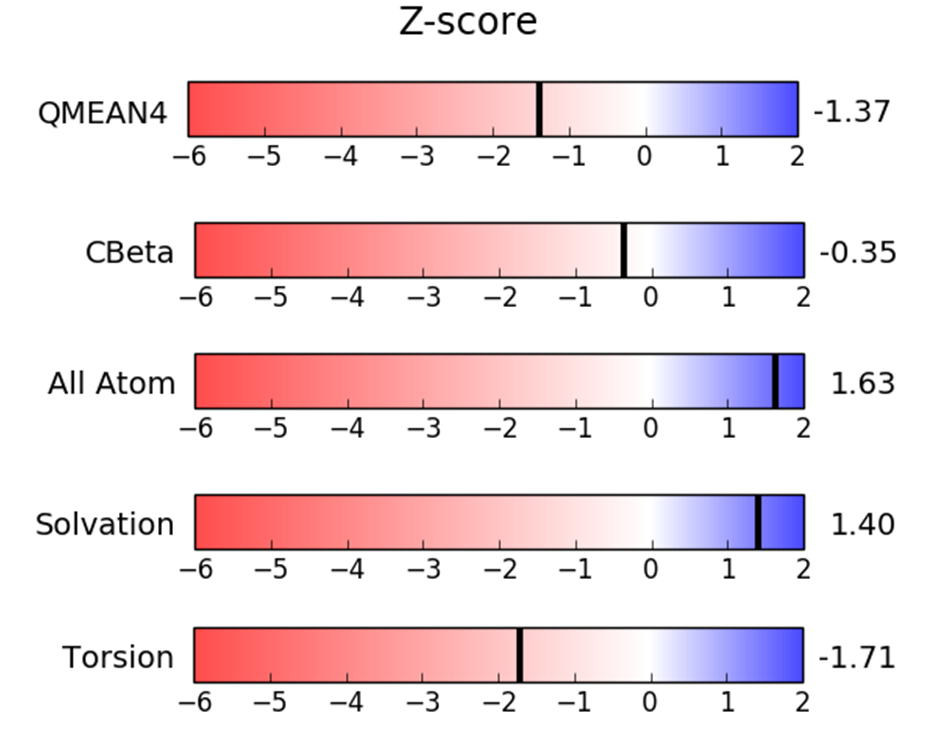
**

**(C) (D)**

**Figure S2. The results of quality evaluation of protein models**

1. QMEAN Z-score of HalI; (B) Normalized QMEAN Score of HalI; (C) QMEAN Z-score of HalR; (D) Normalized QMEAN Score of HalR

**
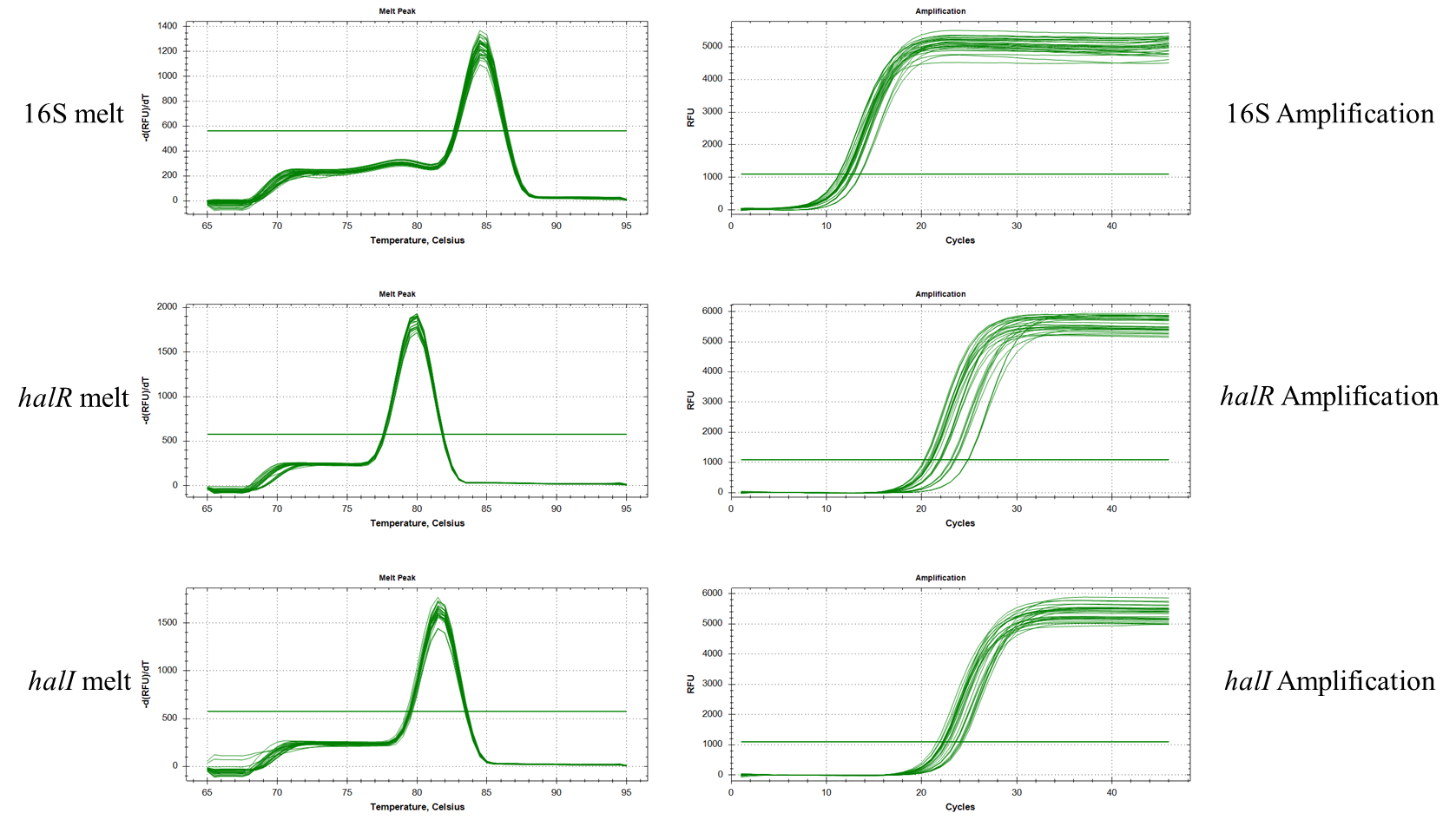
**

**Figure S3. Melt and amplification curves of the target gene**

**Table S1 Inhibitory activity of L-carvone on Swimming and Swarming Motility Distances by *H. alvei* (mean ± standard deviation).**

| Additive | Concentration | Bacterial motility (mm)^a^ | | Inhibitory rate (%)^b^ | | |
| --- | --- | --- | --- | --- | --- | --- |
|  |  | Swimming | Swarming | Swimming | | Swarming |
| C_6_-HSL | 20 μg/mL | 55.43 ± 0.15^c^ | 71.89 ± 0.31^c^ | | — | — |
| Control | 0 μL/mL | 42.17 ± 0.10^d^ | 59.13 ± 0.12^d^ | | — | — |
| L-carvone | 0.0625 μL/mL | 36.93 ± 0.13^e^ | 45.36 ± 0.17^e^ | | 12.43 % | 23.29 % |
| L-carvone | 0.125 μL/mL | 26.87 ± 0.07^f^ | 22.83 ± 0.19^f^ | | 36.28 % | 61.39 % |
| L-carvone | 0.25 μL/mL | 18.29 ± 0.23^g^ | 18.17 ± 0.27^g^ | | 56.63 % | 69.27 % |
| L-carvone | 0.50 μL/mL | 16.24 ± 0.12^g^ | 14.82 ± 0.09^g^ | | 61.49 % | 74.94 % |

a Expressed as migration distance of *H. alvei* on swimming and swarming plates

b The inhibitory rate = (Distance of control group – Distance of treated group) / Distance of control group

c-g Significantly different means (P < 0.05)

***Table S2****. Structures and scores of models of H. alvei Lux-type proteins*

| Proteins | Template | Description | Seq-similarity | GMQE | QMEAN | Model | |
| --- | --- | --- | --- | --- | --- | --- | --- |
| HalI  (LuxI-type) | 1k4j.1.A | acyl-homoserinelactone synthase EsaI | 0.49 | 0.75 | -1.85 | | 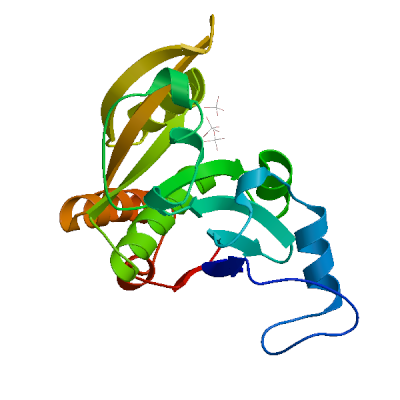 |
|  | 1ro5.1.A | Autoinducer synthesis protein lasI | 0.31 | 0.60 | -2.84 | | 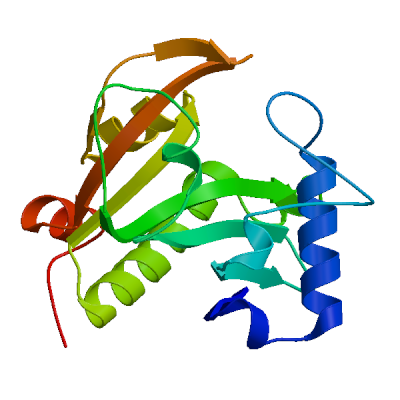 |
|  | 5w8c.1.A | Autoinducer synthase | 0.29 | 0.58 | -3.43 | | 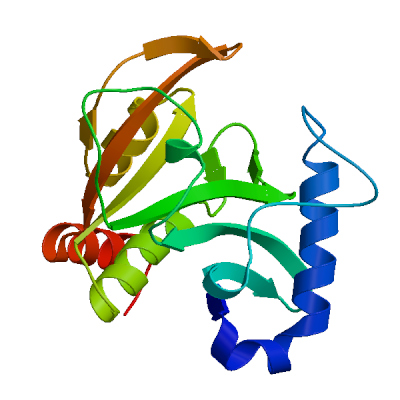 |
| HalR  (LuxR-type) | 5l07.1.B | Quorum-sensing transcriptional activator | 0.41 | 0.51 | -1.37 | | 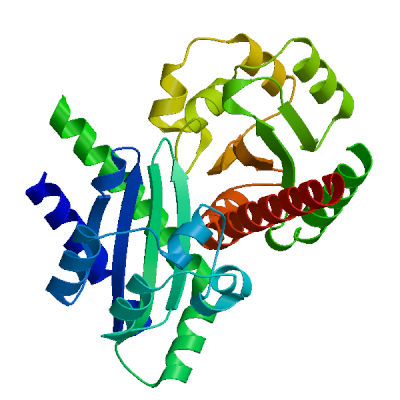 |
|  | 4y15.2.A | Transcriptional regulator of ftsQAZ gene cluster | 0.35 | 0.53 | -4.15 | | 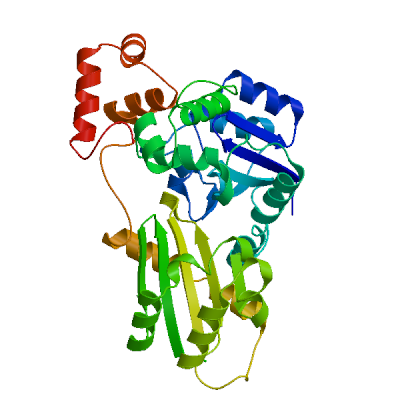 |
|  | 4lgw.1.A | Regulatory protein SdiA | 0.35 | 0.52 | -5.30 | | 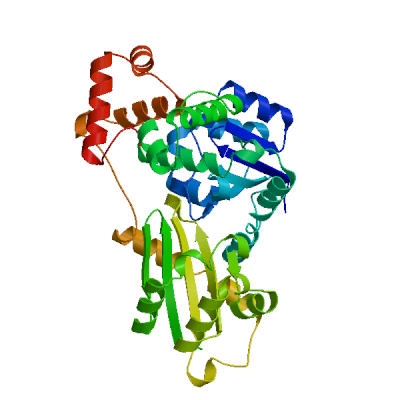 |

**Table S3**. Docking results of the HalI and HalR protein model of *H. alvei* with ligands

| Protein | Ligand | LibDock score ^a^ | Absolute  Energy ^b^ | Relative  Energy ^b^ |
| --- | --- | --- | --- | --- |
| HalI | L-carvone | 71.0676 | 14.6593 | 4.11795 |
| HalR | C_6_-HSL | 84.7765 | 13.5728 | 0.2145 |
|  | L-carvone | 66.7963 | 13.8250 | 3.2836 |
|  | Halogenated furanone C30 | 52.7221 | 17.3224 | 0 |

a LibDock score is the function for ranking the binding affinity of ligands to the active site of a receptor

b Absolute Energy and Relative Energy represents the required energy of docking by the ligand with the protein
